# Association of the efficiency of hemodialysis instruments in the removal of microbial and chemical pollutant

**DOI:** 10.3389/fpubh.2022.947782

**Published:** 2022-09-08

**Authors:** Habib Allah Shahriyari, Abduladheem Turki Jalil, Gholamreza Sarizadeh, Zebuniso R. Shodmonova, Afshin Takdastan, Fatemeh Kiani, Mohammad Javad Mohammadi

**Affiliations:** ^1^School of Public Health, Ahvaz Jundishapur University of Medical Sciences, Ahvaz, Iran; ^2^Medical Laboratories Techniques Department, Al-Mustaqbal University College, Hilla, Iraq; ^3^Environmental Technologies Research Center, Ahvaz Jundishapur University of Medical Sciences, Ahvaz, Iran; ^4^Department of Urology, Samarkand State Medical Institute, Samarkand, Uzbekistan; ^5^Department of Environmental Health Engineering, School of Public Health, Ahvaz Jundishapur University of Medical Sciences, Ahvaz, Iran; ^6^Student Research Committee, Ahvaz Jundishapur University of Medical Sciences, Ahvaz, Iran

**Keywords:** hemodialysis instruments, microbial pollutants, chemical pollutant, water, Iran, kidney failure

## Abstract

Dialysis water is vital because of various harmful contaminants for patients. The aim of this study was to assess the efficiency of hemodialysis instruments in the removal of microbial and chemical pollutant in educational hospitals affiliated to Ahvaz Jundishapur University of medical sciences, Iran during 2018–2019. This cross-sectional descriptive research studied the microbial and chemical water quality of hemodialysis instruments in Razi, Sina, and Golestan hospitals in Ahwaz, Iran. 72 samples of microbial parameters and 24 samples of chemical parameters were collected from water used in hemodialysis instruments, including microbial characteristics (the total coliform, fecal coliform and heterotrophic bacteria counts) and chemical characteristics (pH, turbidity, PO_4_, Cl, Mg, So_4_, Ca, NO_2_, and EC) at Razi, Imam, and Golestan educational hospitals on all weekdays during 2018-2019. In this study, experiments were done according to the current standard methods, EPA from hemodialysis instruments. Finally, using SPSS18 software and descriptive statistics, the relationship between results at the removal of toxic, microbial, and chemical pollutants in different months and hospitals was investigated. this study showed that the average concentration of chemical characteristics during the warm season at Razi, Imam, and Golestan educational hospitals for pH, Turbidity, PO_4_, Cl, Mg, So_4_, Ca, NO_2_, and EC were (6.867, 6.4475, 6.53); (2.985, 3.035, 1.226); (0.075, 0.245, 0.195); (38.5, 21.965, 144.87); (1.552, 1.657, 39.445); (8.6, 4.5, 21.5), (2.09, 3.187, 78.975); (0.0082, 0.038, 0.155), and (125.25, 70.35, 78.35), respectively during 2018. Also, during 2019, results showed that the average levels of amounts for pH, Turbidity, PO_4_, Cl, Mg, So_4_, Ca, NO_2_, and EC in Razi, Imam, and Golestan educational hospitals were (7.077, 7.252, 6.435), (1.725, 0.595, 4.16), (0.0775, 0.0597, 0.0297), (52.33, 138.81, 20.92), (23.52, 18.227, 8.767), (35, 27.25, 4.05), (14.58, 28.152, 9.25), (0.0067, 0.0045, 0.0032), and (210.52, 121.62, 29.16), respectively. According to the results, hemodialysis instruments in Razi and Imam have a 90% efficiency in removing heterotrophic bacteria counts (HPC). Based on these findings, educational hospital hemodialysis equipment effluent in Ahvaz, Iran was mitted to Iran environmental standards for use in hemodialysis machines. The result showed that the removal percentage level of microbial and chemical pollutants by the hemodialysis process is comparatively suitable. It should be mentioned that in the proper operation and reconstruction, hemodialysis systems can have an increased rate of removal of microbial and chemical pollutants.

## Introduction

Levels of microbial and chemical pollutants in water used in hemodialysis machines have become a global problem today for patents (1). Entering these pollutants into the hemodialysis machines is considered a serious threat to humans (1).

Water quality is important for preparation of dialysis solution due to its direct relationship with blood of patients with renal failure (2). Hospital drinking water is one of the most important problems that can affect health staff and patients (3). Pollutants entering the water supply and food chain in various ways are regarded as a serious threat to humans and other organisms (4–6).

The used dialysis liquid is considered as the largest volume of water used in medicine (7). Water used by hemodialysis machines because of the sensitive and dangerous effects it can cause for patients should be complementary treatment and without any contaminating elements (8, 9). Based on results from different studies among dialysis patients, fatal fever-causing reactions, production of endotoxin and infection are the most current complications of microbial contamination of water in hemodialysis instruments (8, 10, 11).

Based on several reported studies, the main chemical agents' effects on process dialysis in hemodialysis machines include trace elements (chromium, lead, cadmium, silver, selenium, cyanide, barium, tin, arsenic); ionic compounds (iron, manganese, nitrate, copper, iodine, chlorine, zinc); chemical additives to water (chloramines, fluoride, aluminum) and physiology elements (sodium, potassium, calcium) (12–18). The process used for water treatment for hemodialysis is reverse osmosis (10, 19). Blood purification instead of kidney defective and clearing toxins in the blood by a permeable membrane is done by using hemodialysis to a permeable membrane that replaces the function of the kidney glomeruli (20–22).

The use of normal tap water always carries the possibility of transferring potentially toxic substances from the dialysis fluid to the patient's blood, therefore the quality of the water used to prepare the dialysis solution is very important. Because of the logical connection between water quality and the health of patients Dialysis, physicochemical and microbial quality compliance water used to prepare dialysis fluid with standards international seem necessary (2).

Pathogenic microorganisms, disinfectants, pharmaceuticals, toxic, radioactive elements, chemicals, and microbial pollutants in water resources can all have a significant impact on human and animal health (23, 24). The most serious pollutant effects on humans are insomnia, infection, phototoxicity, vomiting, diarrhea, dizziness, headache, and shock in kidney patients (25, 26).

The distribution network, state hospital sanitation, number of beds for dialysis, cultural situation, climatic conditions are the main agents affecting the quality and quantity of medical center water used in hemodialysis instruments (10, 19). Evaluating and comparing hospitals' quality drinking water and inlet water to hemodialysis machines is usually done by measuring levels of toxic, coliform bacteria, microbial and chemical pollutants (27–29). Removal of hazardous substances and toxic elements from the blood is the most important role of the dialysis process in patients whose kidney function is impaired (30–32). Evaluating and comparing quality hemodialysis machines is usually done by measuring the levels of toxic, coliform bacteria, heterotrophic bacteria counts (HPC), microbial and chemical elements (1).

Surface waters are the primary source of water in Iran's southwestern region and the Khuzestan Plain (especially Ahvaz). Due to the abundant resources of surface water and fertile soil, this region is one of the most strategic agricultural poles of Iran, which has a large share in the production of agricultural products (33). In Ahvaz, Iran, increasing industrial activities combined with the production of pollutants, including toxic, organic and microbial pollutants, are one of the serious and expanding problems facing humans today (18, 34–37).

The high potential of Ahvaz for development (strategic and industrial activities) and the need to equip and construct new medical centers and hospitals, increases the importance of this research. As a result, the primary goal of this study was to determine the efficacy of hemodialysis instruments in removing toxic, microbial, and chemical pollutants in the educational hospital Ahvaz Jundishapur University of Medical Sciences in southwest Iran from 2018 to 2019.

## Materials and methods

### Site of study

**Figure 1:**
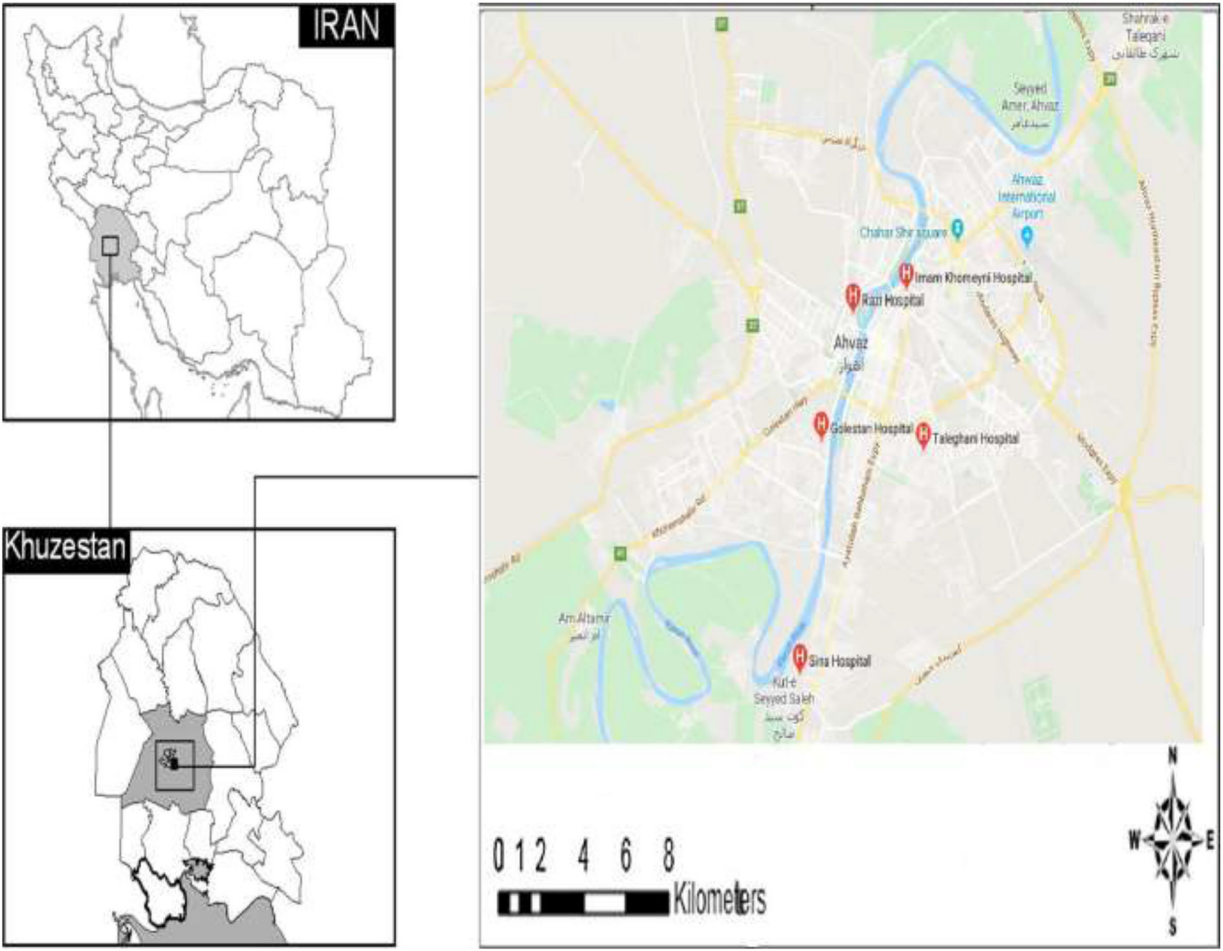
Location of the study area educational hospital affiliated to Ahvaz Jundishapur University of medical sciences, in the south west of Ahvaz, Iran.

This cross-sectional descriptive research studied the toxic, microbial, and chemical water quality of hemodialysis instruments in Razi, Imam, and Golestan educational hospitals of Ahvaz (located in south-western Iran). It borders Iraq on the west and the Persian Gulf on the south (Figure 1) (38). Ahvaz Jundishapur University of medical sciences has six medical training centers, and in this study we selected three educational hospitals due to the volume of referrals and the fact that they have the most dialysis beds. Razi, Imam and Golestan educational hospital are a tertiary-care hospital with 220, 810 and 718 beds, located in Ahvaz (39–41) (Figure 1). pH, SO_4_, Na, Ca, Mg, and HPC values were determined according to the standard methods (42–45).

### Sampling and collected data

In this study, we used to assess the potential haemodialysis devices on removing the microbial characteristics (the total coliform, fecal coliform and heterotrophic bacteria counts) and chemical characteristics (pH, Turbidity, PO_4_, Cl, Mg, So_4_, Ca, NO_2_, and EC) in educational hospital affiliated to Ahvaz Jundishapur University of medical sciences (located in south-western Iran) during 2018–2019 by using hemodialysis devices system which includes 2 years including 2018–2019.

Twenty four stages of sampling were collected (3 stages in each month simple random sampling on different days' week and during the activity of hemodialysis machines (at 8:00 a.m. to 14 PM in each hospital) in sterile glass containers with a sanding head to volume 250 mL from the water inlet to hemodialysis machines. Samples of input hemodialysis devices were collected from Razi, Imam, and Golestan hospitals' dialysis departments and transferred to a laboratory for further analysis. Temperature and pH parameters were measured *in situ*. In this study, 72 samples were collected. In this study, we measured the total coliform, fecal coliform, and heterotrophic bacteria counts (CFU; Colony Forming Unit), residual chlorine, sodium (flame photo meter), magnesium, calcium (titration), and sulfate (spectrophotometry method) on the efficiency of removal (46–48). Electrical conductivity (EC) and pH measurement by using a (HQ40d) analyzer. Finally, the relationship between results at different months and stations was investigated using SPSS and descriptive statistics.

### Statistical analysis

The levels of microbial and chemical pollutants in hemodialysis instruments were collected from the three hospitals in Ahvaz city (Razi, Imam, and Golestan educational hospital) during 2018–2019. The coded data was entered into the SPSS software. Data analyses were performed, using SPSS-18. The data were analyzed using descriptive statistics and the mean standard deviation of the mean (SD).

## Results

In this study, we evaluated the microbial and chemical quality of water entering the hemodialysis devices of Razi, Imam, and Golestan educational hospitals in a period of one year in 2018–2019. The efficiency studies of pollutants' removal by hemodialysis instruments were done at different characteristics. Supplementary Table 1 shows the factors which affected the influent component, including total coliform and fecal coliform, HPC, turbidity, pH, PO_4_, Cl, Mg, So_4_, Ca, NO_2_, and EC characteristics of the hospital hemodialysis machines (Supplementary Table 1).

**Figure 2:**
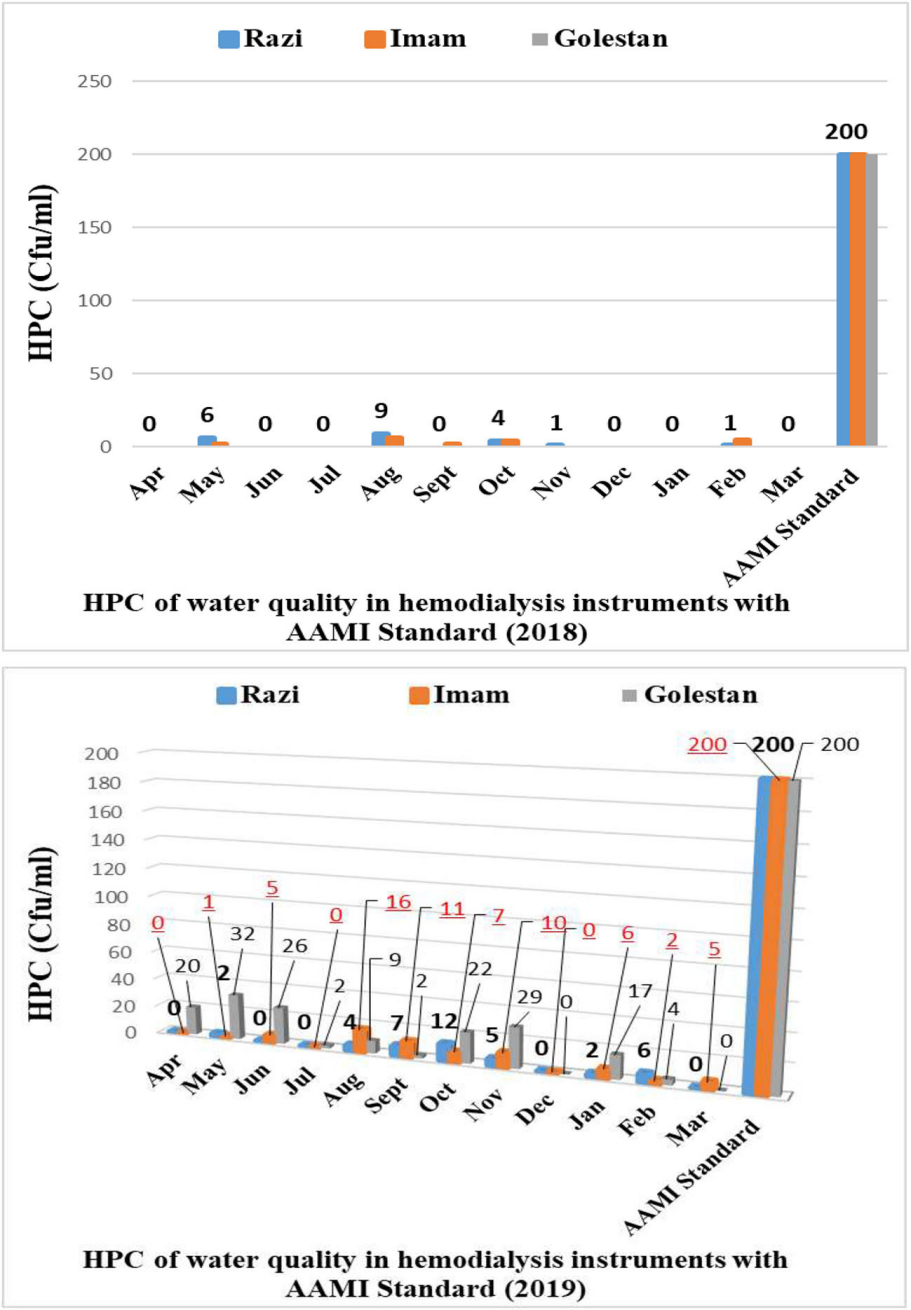
The annual average concentration of heterotrophic plate count during 2018–2019.

According result of this study the level of total coliform and fecal coliform in during years 2018 and 2019 were zero (Supplementary Table 1). The result of heterotrophic plate count (HPC) in during 2018-2019 express in Figure 2. Supplementary Table 1 showed that average concentration of HPC in Razi, Imam and Golestan hospital in 2018 and 2019 were (1.75, 1.583, 0 Cfu/ml) and (3.166, 5.25, 13.583 Cfu/ml), respectively.

**Figure 3:**
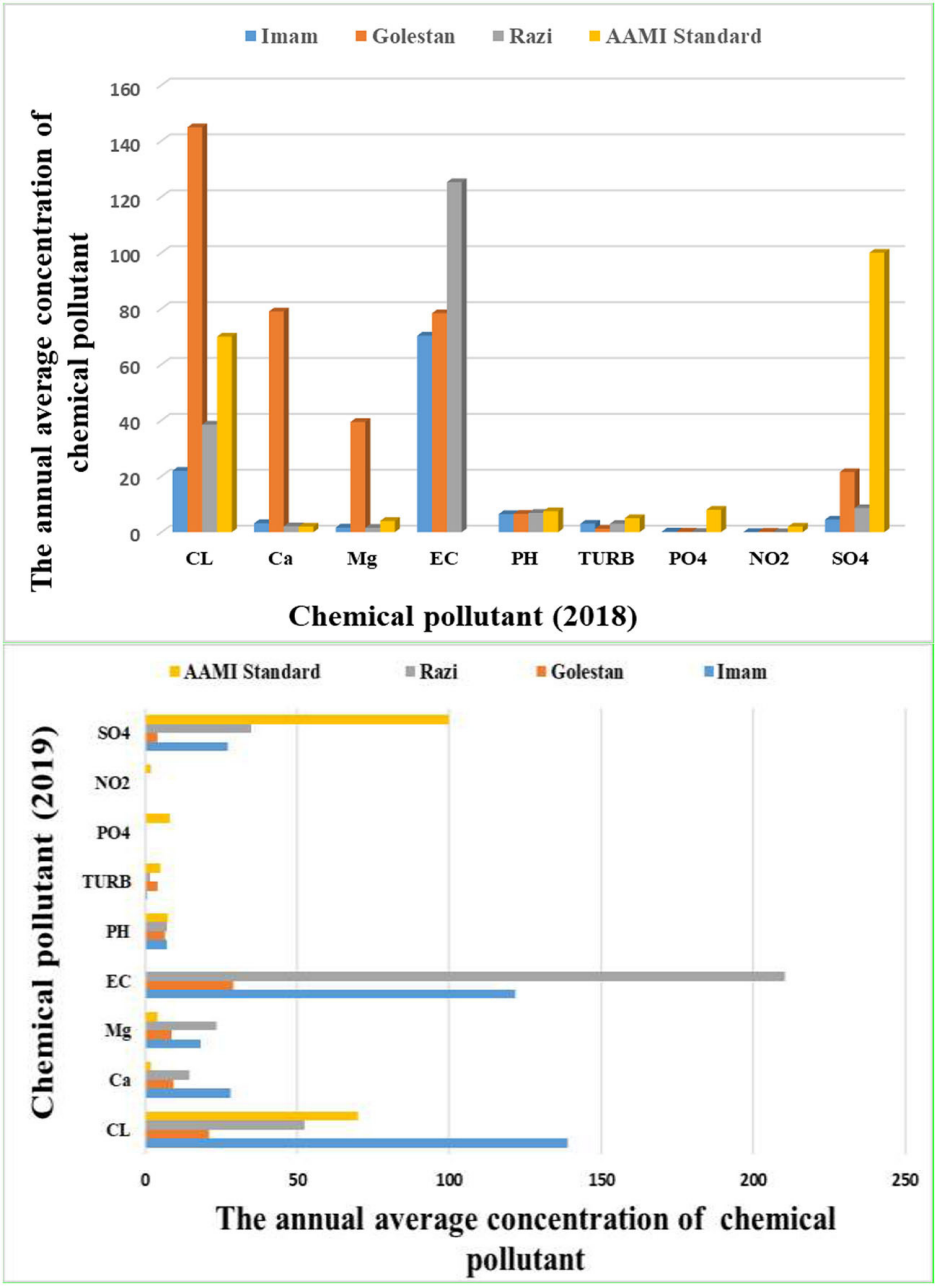
The annual average concentration of chemical pollutant during 2018–2019.

Figure 3 showed that in Razi, Imam and Golestan educational hospital in during 2018, average concentration of pH, Turbidity, PO_4_, Cl, Mg, So_4_, Ca, NO_2_, and EC were (6.867, 6.4475, 6.53.2), (2.985, 3.035, 1.226), (0.075, 0.245, 0.195), (38.5, 21.965, 144.87), (1.552, 1.657, 39.445), (8.6, 4.5, 21.5), (2.09, 3.187, 78.975), (0.0082, 0.038, 0.155), and (125.25, 70.35, 78.35), respectively. Figure 3 showed that in Razi, Imam and Golestan educational hospital in during 2019, average concentration of pH, Turbidity, PO_4_, Cl, Mg, So_4_, Ca, NO_2_, and EC were (7.077, 7.2525, 6.435), (1.725, 0.595, 4.16), (0.0775, 0.0597, 0.0297), (52.33, 138.81, 20.92), (23.52, 18.227, 8.767), (35, 27.25, 4.05), (14.58, 28.152, 9.25), (0.0067, 0.0045, 0.0032), and (210.52, 121.62, 29.16), respectively.

In 2018, the average concentration of chemical characteristics during the warm season at Razi, Imam, and Golestan educational hospitals for pH, Turbidity, PO_4_, Cl, Mg, So_4_, Ca, NO_2_, and EC were (7.18, 6.305, 7.145), (4.1, 2.99, 0.662), (0.08, 0.34, 0.35), (24.35, 23.06, 245.02), (0.53, 1.14, 60.59), (8.2, 3, 43), (1.63, 2.94, 107.95), (0.012, 0.055, 0.31), and (168, 78.5, 101), respectively (Supplementary Table 1). Also, the mean level of pH, turbidity, PO_4_, Cl, Mg, So4, Ca, NO_2_, and EC during the cold season were (6.555, 6.59, 5.92), (1.87, 3.08, 1.79), (0.07, 0.15, 0.04), (52.65, 20.87, 44.73), (2.575, 2.175, 18.3), (9, 6, 0), (2.55, 3.435, 50), (0.0045, 0.021, 0.001), and (82.25, 62.2, 55.7), respectively during 2018 (Supplementary Table 1).

The result of Supplementary Table 1 showed that in Razi, Imam and Golestan educational hospital in during the warm season 2019, average concentration of pH, Turbidity, PO_4_, Cl, Mg, So_4_, Ca, NO_2_, and EC were (6.645, 7.355, 5.995), (2.95, 0.82, 2.185), (0.07, 0.075, 0.0155), (63.025, 167.06, 28.335), (36.775, 9.255, 6.375), (24.5, 30.5, 5), (27.5, 7.905, 6.3), (0.005, 0.0045, 0.004), and (207.65, 146.5, 43.35), respectively (Supplementary Table 1). Also, Supplementary Table 1 indicate that during the cold season 2019 at Razi, Imam and Golestan hospital the mean concentration of pH, Turbidity, PO_4_, Cl, Mg, So_4_, Ca, NO_2_, and EC were (7.51, 7.15, 6.875), (0.495, 0.37, 6.135), (0.085, 0.0445, 0.044), (41.645, 110.56, 13.505), (10.305, 27.2, 11.16), (45.5, 24, 3.1), (1.66, 48.4, 12.2), (0.0085, 0.005, 0.0025), and (213.4, 96.755, 14.98), respectively during cold season (Supplementary Table 1).

## Discussion

In this study, we evaluated the efficiency of removal of microbial and chemical pollutants in hemodialysis instruments in an educational hospital affiliated to Ahvaz Jundishapur University of medical sciences, Iran during 2018–2019.

According result of this study the level of total coliform and fecal coliform in during years 2018 and 2019 were zero (Supplementary Table 1). Supplementary Table 1 showed that average concentration of HPC in Razi, Imam and Golestan hospital in 2018 and 2019 were (1.75, 1.583, 0 Cfu/ml) and (3.166, 5.25, 13.583 Cfu/ml), respectively. Results of this study demonstrated that total and fecal coliforms and HPC were in accordance with Association for the Advancement of Medical Instrumentation (AAMI) standards that indicate good performance in reverse osmosis processes. Based on the results of this study in Figure 2, higher than 90 percent of heterotrophic bacteria counts (HPC) were removed in Imam and Razi hospitals by hemodialysis instruments that can be demonstrated to be efficient and up-to-date devices. This indicates the proper inspection of the hemodialysis instruments and reverse osmosis (RO) systems used in these hospitals.

In 2011 in Kashan, Iran, Baseri et al. (49) reported that the none of the samples had signs of total coliform, fecal coliform and heterotrophic bacteria counts contamination. In another study, in Qom province hospitals studied performed by Asadi et al. (50). They demonstrated microbial contaminated in the influent water of dialysis machines with AAMI standards (50). The result of our study was same to these studies which can be related to good efficiency hemodialysis instruments and purification process. Also, in Egypt, Ibrahim evaluation the level of microbial and chemical parameters in dialysis units. According to the result, the microbial quality was up to standard (46). Vorbeck-Meister et al. (51) in their studied showed that the microbial quality of water used for haemodialysis were less than the standard amount. Good efficiency hemodialysis instruments can be reasons same the result of our study with these studies.

According to the results of this study, the mean levels of pH, turbidity, PO_4_, Cl, Mg, So_4_, Ca, NO_2_, and EC were (6.867, 6.4475, 6.53), (2.985, 3.035, 1.226), (0.075, 0.245, 0.195), (38.5, 21.965, 144.87), (1.552, 1.657, 39.445), (8.6, 4.5, 21.5), (2.09, 3.187, 78.975), (0.0082, 0.038, 0.155), and (125.25, 70.35, 78.35), respectively during the warm season in Razi, Imam, and Golestan hospitals during 2018 (Figure 3). Also, based on the results of our study in 2019, the concentration of pH, turbidity, PO_4_, Cl, Mg, So_4_, Ca, NO_2_, and EC in Razi, Imam and Golestan educational hospital were (7.077, 7.252, 6.435), (1.725, 0.595, 4.16), (0.0775, 0.0597, 0.0297), (52.33, 138.81, 20.92), (23.52, 18.227, 8.767), (35, 27.25, 4.05), (14.58, 28.152, 9.25), (0.0067, 0.0045, 0.0032), and (210.52, 121.62, 29.16), respectively (Figure 3).

Results showed that Razi, Imam, and Golestan hospital hemodialysis instruments have good efficiency in the removal of toxic, microbial, and chemical pollutants.

Figure 3 showed that NO_2_, PO_4_, turbidity, Mg, and Ca had the maximum removal efficiency by hemodialysis instruments during 2018-2019 in Razi, Imam, and Golestan hospitals.

The sources of chemical contaminants of water (aluminum, chloramine, copper, fluoride, nitrate, sulfate, and zinc) and dialysate with particular toxicity in hemodialysis patients are raw water and municipal water, municipal water, dialysis facility, municipal water, raw water, and dialysis facility, respectively (52–54).

According to different studies, the principal toxicities of chemical contaminants including copper, sulfate, chloramine, aluminum, fluoride, and nitrate are anemia (nausea, vomiting, acidosis), anemia (bone disease, encephalopathy syndrome), cardiovascular disease, and anemia, respectively (52–55).

Baseri et al. (49) studied the water quality of the hemodialysis instruments in Kashan Akhavan hospital. They reported that the none of the samples had signs of bacterial contamination. Also, in another study, Asadi et al. (50) demonstrated heavy metals in the influent water of dialysis machines in Qom province hospitals with AAMI standards. The concentrations of NH_3_, SO_4_, and Na shown by them (50), which is consistent with our results, can be attributed to the same water source and purification process.

Based on a study conducted by Kawanishi et al. (56) in Japan on the new standard of fluids for water entering the hemodialysis devices. According to the obtained results, treatment of heavy metals should be considered because of the effects these pollutants have on patents' health (56). In Hospitals of 22 Bahman Gonabad, Iran Asadzadeh et al. (57) studied the chemical quality of water entering dialysis machines and its comparison with standards. The results of this study showed the measured values were less than standard reference values. In all samples, a fluoride, nitrate, potassium, and sodium were less than standard, but only temporary calcium concentrations expressing difficulty were higher than standard levels (57).

In another study which was done by Marjani et al. (58) in Gorgan, Iran, investigating chemical pollutant levels in hemodialysis patients. Based on the results, the chemical and biological quality that were measured, with the exception of calcium and magnesium, were less than the standard.

Accordingly, a study conducted by Vorbeck-Meister investigated the quality of water used for haemodialysis (bacteriological and chemical parameters). The result of their study showed that the residual chlorine and PH of water were less than the standard amount (51). In a similar study, Ibrahim in Cairo, Egypt, assessed the quality of care and adherence to the international guidelines considering dialysis water treatment in university hospital based dialysis units. Based on the result pH neutral and some elements in the range had exceeded the standard range (46).

Some differences in the efficiency of hemodialysis instruments in the removal of microbial and chemical pollutants can be attributed to the quality of water supplied to water sources (surface and groundwater), the quality of water transmission and distribution network in different regions, geographical and climatic conditions in different regions of Iran or other countries, and the use of modern and up-to-date hemodialysis instruments.

Different result of studies in the field of performance hemodialysis Instruments in dialysis ward at hospital and compared with our findings showed in Table 1.

**Table 1:** The microbiological quality of water used for hemodialysis instruments and comparison of various studies.

**Parameters**	**Hetertrophic plate count (Cfu/ml)**	**Total coliform [(MPN/100 ml L); Cfu/100 ml]**	**Fecal coliform [(MPN/100 ml L); Cfu/100 ml]**
**Study**			
Amira in Egypt (52)	12	0	0
Al-Haik in Hadhramaut-Yemen (59)	3.41	<1.1	<1.1
Soltani et al. in Ahvaz-Iran (1).	1	0	0
Alizadeh et al. in Zahedan-Iran (60)	1	0	0
Asadi et al. in Qom-Iran (61)	0	<1.1	<1.1
Abbaszadeh et al. in East Azerbaijan-Iran (2)	0	<1.1	<1.1
Present study Imam	≈ <3	<1.1	<1.1
Razi	≈ <2	<1.1	<1.1
Golestan	≈ <6	<1.1	<1.1

An increasing amount of chemical and microbial pollutants can contaminate the burden of organic, microbial, chemical, and toxic pollution in the environment of the Karun river and underground sources that are fed from the Karun basin. It should be noted that the reduction of surface and groundwater quality in the region can have a direct effect on water quality in the provision of hemodialysis instruments and kidney patients referred to the dialysis wards of hospitals in Ahvaz.

Any discharge of effluents and pollutants into the water supply sources of citizens and patients can greatly increase the level of dangerous and toxic pollutants that threaten the health of patients.

## Conclusion

This study investigated the performance of efficiency for hemodialysis instrument treatment in the removal of a level of microbial and chemical pollutants. Based on the results of the analysis, microbial and chemical pollutant removal during the study, had a significant direct effect on the river purification with hemodialysis instrument efficiency. Toxic, microbial, and chemical pollutants in the hemodialysis process can be very threatening to the patents. According to the results of this study, the mean value of microbial pollutants (the total coliform, fecal coliform, and heterotrophic bacteria counts) was lower than the AAMI standard value. Also, the average level of chemical pollutants (Ca, Mg, and Cl) was higher than the AAMI standard value. The results of this study showed that pollutants discharged into the Ahvaz Karun river are the main cause of chemical and microbial pollutants in water in this region. The results of this study showed that the health of dialysis patients, the general health of the community, and the environment are directly affected by the essential elements of bioavailability of water. Appropriate microbial and chemical water quality used in dialysis machines can be referred to the use of appropriate devices, periodic monitoring and supervision of medical engineering and environmental health experts, proper management of these devices, and the importance of managers to provide optimal services to kidney patients in the dialysis ward. The most important trends that health policymakers and experts should pay attention are updating dialysis machines with new technology, using new membrane filters with higher efficiency and using primary sources of higher quality water among. Further studies are required to assess the efficiency of hemodialysis instruments in the removal of the level of another pollutant that threatens the patents' health.

## Data availability statement

The original contributions presented in the study are included in the article/Supplementary materials, further inquiries can be directed to the corresponding authors.

## Author contributions

HAS, ATJ, GS, ZS, AT, FK, and MJM were principal investigators of the study, drafted the manuscript, and performed the statistical analysis. HAS, ATJ, GS, and MJM were advisors of the study. All authors contributed to the design and data analysis, assisted in the preparation of the final version of the manuscript, read, and approved the final version of the manuscript.

## Funding

This work was funded by the grant: (ETRC-9907) from Ahvaz Jundishapur University of Medical Sciences. This study was originally approved by the Ahvaz Jundishapur University of Medical Sciences with code IR.AJUMS.REC.1399.512.

## Conflict of interest

The authors declare that the research was conducted in the absence of any commercial or financial relationships that could be construed as a potential conflict of interest.

## Publisher's note

All claims expressed in this article are solely those of the authors and do not necessarily represent those of their affiliated organizations, or those of the publisher, the editors and the reviewers. Any product that may be evaluated in this article, or claim that may be made by its manufacturer, is not guaranteed or endorsed by the publisher.
