# Supplementary material for: Association of the efficiency of hemodialysis instruments in the removal of microbial and chemical pollutant
Source: Front Public Health. 2022 Sep 8;10:947782. doi: 10.3389/fpubh.2022.947782 (PMC9496643; doi:10.3389/fpubh.2022.947782)
Supplement: Supplementary file 1 [file Table_1.doc]

**Table S1**. The average chemical and biological characteristics of water quality in Razi, Imam and Golestan hospital hemodialysis instruments

| parameter | **Apr** | **May** | **Jun** | **Jul** | **Aug** | **Sept** | **Oct** | **Nov** | **Dec** | **Jan** | **Feb** | **Mar** | Annual average±SD | AAMI Standard |
| --- | --- | --- | --- | --- | --- | --- | --- | --- | --- | --- | --- | --- | --- | --- |
| Total coliforms (MPN/100 ml L)*  2018 | 0 | 0 | 0 | 0 | 0 | 0 | 0 | 0 | 0 | 0 | 0 | 0 | 0 | <1.1 |
| 2019 | 0 | 0 | 0 | 0 | 0 | 0 | 0 | 0 | 0 | 0 | 0 | 0 | 0 |
| Total coliforms (MPN/100 ml L)**  2018 | 0 | 0 | 0 | 0 | 0 | 0 | 0 | 0 | 0 | 0 | 0 | 0 | 0 |
| 2019 | 0 | 0 | 0 | 0 | 0 | 0 | 0 | 0 | 0 | 0 | 0 | 0 | 0 |
| Total coliforms (MPN/100 ml L)***  2018 | 0 | 0 | 0 | 0 | 0 | 0 | 0 | 0 | 0 | 0 | 0 | 0 | 0 |
| 2019 | 0 | 0 | 0 | 0 | 0 | 0 | 0 | 0 | 0 | 0 | 0 | 0 | 0 |
| Fecal coliform (MPN/100 ml L)*  2018 | 0 | 0 | 0 | 0 | 0 | 0 | 0 | 0 | 0 | 0 | 0 | 0 | 0 | <1.1 |
| 2019 | 0 | 0 | 0 | 0 | 0 | 0 | 0 | 0 | 0 | 0 | 0 | 0 | 0 |
| Fecal coliform (MPN/100 ml L)**  2018 | 0 | 0 | 0 | 0 | 0 | 0 | 0 | 0 | 0 | 0 | 0 | 0 | 0 |
| 2019 | 0 | 0 | 0 | 0 | 0 | 0 | 0 | 0 | 0 | 0 | 0 | 0 | 0 |
| Fecal coliform (MPN/100 ml L)***  2018 | 0 | 0 | 0 | 0 | 0 | 0 | 0 | 0 | 0 | 0 | 0 | 0 | 0 |
| 2019 | 0 | 0 | 0 | 0 | 0 | 0 | 0 | 0 | 0 | 0 | 0 | 0 | 0 |
| HPC (Cfu/ml)*  2018 | 0 | 6 | 0 | 0 | 9 | 0 | 4 | 1 | 0 | 0 | 1 | 0 | 1.75 ± 0.41 | <200 |
| 2019 | 0 | 2 | 0 | 0 | 4 | 7 | 12 | 5 | 0 | 2 | 6 | 0 | 3.166 ± 0.3 |
| HPC (Cfu/ml)**  2018 | 0 | 2 | 0 | 0 | 6 | 2 | 4 | 0 | 0 | 0 | 5 | 0 | 1.583 ± 0.23 |
| 2019 | 0 | 1 | 5 | 0 | 16 | 11 | 7 | 10 | 0 | 6 | 2 | 5 | 5.25 ± 0.65 |
| HPC (Cfu/ml)***  2018 | 0 | 0 | 0 | 0 | 0 | 0 | 0 | 0 | 0 | 0 | 0 | 0 | 0 |
| 2019 | 20 | 32 | 26 | 2 | 9 | 2 | 22 | 29 | 0 | 17 | 4 | 0 | 13.583 ± 1.3 |
| **Chemical characteristics** | Average concentration of chemical characteristics during warm season | | | | | | Average concentration of chemical characteristics during cold season | | | | | | The annual average concentration of chemical characteristics | AAMI Standard |
| pH*  2018 | 7.18 | | | | | | 6.555 | | | | | | 6.867 ± 0.68 | 6.5-9 |
| 2019 | 6.645 | | | | | | 7.51 | | | | | | 7.077 ± 0.87 |
| pH**  2018 | 6.305 | | | | | | 6.59 | | | | | | 6.4475 ± 0.68 |
| 2019 | 7.355 | | | | | | 7.15 | | | | | | 7.2525 ± 0.59 |
| pH***  2018 | 7.145 | | | | | | 5.92 | | | | | | 6.53.2 ± 0.64 |
| 2019 | 5.995 | | | | | | 6.875 | | | | | | 6.435 ± 0.71 |
| Turbidity (NTU)*  2018 | 4.1 | | | | | | 1.87 | | | | | | 2.985 ± 0.42 | <5 |
| 2019 | 2.95 | | | | | | 0.495 | | | | | | 1.725 ± 0.2 |
| Turbidity (NTU)**  2018 | 2.99 | | | | | | 3.08 | | | | | | 3.035 ± 0.41 |
| 2019 | 0.82 | | | | | | 0.37 | | | | | | 0.595 ± 0.12 |
| Turbidity (NTU)***  2018 | 0.6625 | | | | | | 1.79 | | | | | | 1.226 ± 0.6 |
| 2019 | 2.185 | | | | | | 6.135 | | | | | | 4.16 ± 0.85 |
| PO_4_ (mg/l)*  2018 | 0.08 | | | | | | 0.07 | | | | | | 0.075 ± 0.004 | <8 |
| 2019 | 0.07 | | | | | | 0.085 | | | | | | 0.0775 ± 0.005 |
| PO_4_ (mg/l)**  2018 | 0.34 | | | | | | 0.15 | | | | | | 0.245 ± 0.08 |
| 2019 | 0.075 | | | | | | 0.0445 | | | | | | 0.0597 ± 0.02 |
| PO_4_ (mg/l)***  2018 | 0.35 | | | | | | 0.04 | | | | | | 0.195 ± 0.025 |
| 2019 | 0.0155 | | | | | | 0.044 | | | | | | 0.0297 ± 0.008 |
| Cl (mg/l)*  2018 | 24.35 | | | | | | 52.65 | | | | | | 38.5 ± 5.7 | <70 |
| 2019 | 63.025 | | | | | | 41.642 | | | | | | 52.33 ± 9 |
| Cl (mg/l)**  2018 | 23.06 | | | | | | 20.87 | | | | | | 21.965 ± 3.46 |
| 2019 | 167.06 | | | | | | 110.56 | | | | | | 138.81 ± 18.4 |
| Cl (mg/l)***  2018 | 245.02 | | | | | | 44.73 | | | | | | 144.87 ± 21 |
| 2019 | 28.335 | | | | | | 13.505 | | | | | | 20.92 ± 3.5 |
| Mg (mg/l)*  2018 | 0.53 | | | | | | 2.575 | | | | | | 1.552 ± 0.09 | <4 |
| 2019 | 36.775 | | | | | | 10.305 | | | | | | 23.52 ± 2.3 |
| Mg (mg/l)**  2018 | 1.14 | | | | | | 2.175 | | | | | | 1.657 ± 0.78 |
| 2019 | 9.255 | | | | | | 27.2 | | | | | | 18.227 ± 2.31 |
| Mg (mg/l)***  2018 | 60.59 | | | | | | 18.3 | | | | | | 39.445 ± 6.2 |
| 2019 | 6.375 | | | | | | 11.16 | | | | | | 8.767 ± 0.72 |
| So_4_ (mg/l)*  2018 | 8.2 | | | | | | 9 | | | | | | 8.6 ± 1.63 | <100 |
| 2019 | 24.5 | | | | | | 45.5 | | | | | | 35 ± 9.5 |
| So_4_ (mg/l)**  2018 | 3 | | | | | | 6 | | | | | | 4.5 ± 1.07 |
| 2019 | 30.5 | | | | | | 24 | | | | | | 27.25 ± 4.28 |
| So_4_ (mg/l)***  2018 | 43 | | | | | | 0 | | | | | | 21.5 ± 10 |
| 2019 | 5 | | | | | | 3.1 | | | | | | 4.05 ± 1.2 |
| Ca (mg/l)*  2018 | 1.63 | | | | | | 2.55 | | | | | | 2.09 ± 0.2 | <2 |
| 2019 | 27.5 | | | | | | 1.66 | | | | | | 14.58 ± 2.3 |
| Ca (mg/l)**  2018 | 2.94 | | | | | | 3.435 | | | | | | 3.187 ± 0.69 |
| 2019 | 7.905 | | | | | | 48.4 | | | | | | 28.152 ± 5.08 |
| Ca (mg/l)***  2018 | 107.95 | | | | | | 50 | | | | | | 78.975 ± 13.5 |
| 2019 | 6.3 | | | | | | 12.2 | | | | | | 9.25 ± 1.07 |
| NO_2_ (mg/l)*  2018 | 0.012 | | | | | | 0.0045 | | | | | | 0.0082 ± 0.0001 | <2 |
| 2019 | 0.005 | | | | | | 0.0085 | | | | | | 0.0067 ± 0.00012 |
| NO_2_ (mg/l)**  2018 | 0.055 | | | | | | 0.021 | | | | | | 0.038 ± 0.004 |
| 2019 | 0.0045 | | | | | | 0.005 | | | | | | 0.0045 ± 0.0002 |
| NO_2_ (mg/l)***  2018 | 0.31 | | | | | | 0.001 | | | | | | 0.155 ± 0.04 |
| 2019 | 0.004 | | | | | | 0.0025 | | | | | | 0.0032 ± 0.0006 |
| EC(μS/cm)*  2018 | 168 | | | | | | 82.25 | | | | | | 125.25 ± 12.9 | 100 |
| 2019 | 207.65 | | | | | | 213.4 | | | | | | 210.52 ± 30.6 |
| EC(μS/cm)**  2018 | 78.5 | | | | | | 62.2 | | | | | | 70.35 ± 13.5 |
| 2019 | 146.5 | | | | | | 96.755 | | | | | | 121.62 ± 28 |
| EC(μS/cm)***  2018 | 101 | | | | | | 55.7 | | | | | | 78.35 ± 18.25 |
| 2019 | 43.35 | | | | | | 14.98 | | | | | | 29.16 ± 7.2 |
| * Razi  ** Imam  *** Golestan  AAMI: Association for the Advancement of Medical Instrumentation (mg/l (meq/l))  EC: Electrical Conductivity  TDS: Tot al Dissolved Solids  HPC: Heterotrophic plate count | | | | | | | | | | | | | | |
